# Efficacy of pulmonary transplantation of engineered macrophages secreting IL-4 on acute lung injury in C57BL/6J mice

**DOI:** 10.1038/s41419-019-1900-y

**Published:** 2019-09-11

**Authors:** Huiying Liu, Yuan He, Cheng Lu, Pengfei Zhang, Chenchen Zhou, Yanli Ni, Wenkai Niu, Xin Yuan, Puyuan Li, Jing Zheng, Yanhong Qin, Luo Zhang, Changqing Bai

**Affiliations:** 1 Department of Respiratory and Critical Care Diseases, The Fifth Medical Center of PLA General Hospital, 100071 Beijing, China; 2 Department of Biomedical Engineering, The Fifth Medical Center of PLA General Hospital, 100071 Beijing, China; 3 Laboratory of Oncology, The Fifth Medical Center of PLA General Hospital, 100071 Beijing, China; 4 Department of Respiratory and Critical Care Diseases, General Hospital of Shenzhen University, 518051 Shenzhen, China

**Keywords:** Bone marrow transplantation, Acute inflammation

## Abstract

Acute lung injury (ALI) and acute respiratory distress syndrome (ARDS) are major causes of respiratory failure, but currently, no effective pharmacotherapy exists for these disorders. Alveolar macrophages play a critical role in both the acute/initial phase and chronic/resolving phase of ALI, rendering them a potential therapeutic target. Interleukin-4 (IL-4), a Th2 cytokine, not only directly inhibits the secretion of pro-inflammatory factors from macrophages but also drives macrophages to the anti-inflammatory and tissue remodeling M2 type. However, the short half-life of IL-4 in vivo hampers its effect on disease treatment. In this study, macrophages secreting IL-4 (M-IL-4) were established and used to treat ALI through pulmonary macrophage transplantation (PMT). The results showed that highly sustained levels of IL-4 and M2 macrophage markers were detected in mice lungs following pulmonary M-IL-4 transplantation. Furthermore, PMT improved the therapeutic effect by reducing lung inflammation, alleviating tissue injury, reducing alveolar macrophages necrotic cell death, and decreasing mortality in mice with ALI. These results suggest an efficient macrophage-based protein drug delivery strategy, and for the first time, prove the feasibility and efficacy of PMT in ALI treatment.

## Introduction

Acute lung injury (ALI) and its severe form, known as acute respiratory distress syndrome (ARDS)^1^, are caused by direct pulmonary insult and indirect systemic inflammatory responses that result from conditions such as sepsis, trauma, and major surgery^1,2^. ALI or ARDS and failure to resolve ongoing inflammation increase the mortality and morbidity of survivors^3^. Despite a large number of researches to understand the pathogenesis of ALI and ARDS, no effective pharmacotherapy has been reported^4,5^. ALI is characterized by alveolar barrier disruption and lung inflammation^6^. Two phases of inflammatory response have been recognized in an ALI animal model. The acute/initial phase is characterized by local vasodilation, increased capillary permeability, and release of inflammatory mediators including histamine, serotonin, and prostaglandins. In contrast, the chronic/resolving phase is identified by the infiltration of leukocytes and phagocytic cells^7,8^.

Alveolar macrophages play critical roles in both phases of ALI^9^. In the acute phase, alveolar macrophages polarize to the classical (M1) activation phenotype, initiate an inflammatory response, and promote neutrophil infiltration and tissue damage^10^. Classically activated or M1 macrophages express high levels of pro-inflammatory cytokines such as tumor necrosis factor-α (TNF-α), interleukin-6 (IL-6), IL-1β, and interferon-γ (IFN-γ), which critically contribute to pathogen elimination^11^. In contrast, alveolar macrophages in the resolving phase exhibit an alternative (M2) activation phenotype, secrete anti-inflammatory factors such as IL-10, IL-1ra, and transforming growth factor beta (TGF-β), and contribute to inflammatory resolution and tissue remodeling^11,12^. Therefore, inhibiting M1 activation of alveolar macrophages in the acute phase and promoting M2 activation in the resolving phase could be an effective therapeutic strategy^13^.

The cytokine IL-4 induces host defense responses against helminth infections and M2 polarization of macrophages in a STAT6-dependent manner^14^. It has been reported that IL-4 treatment decreases mortality and accelerates the resolution of ALI^15^. In some studies, IL-4 was complexed with an anti-IL-4 antibody to prolong its bioavailability, extending its half-life in mice from 0.5 to 24 h; however, injection of an IL-4 intratracheal complex had to be performed each day^15,16^. IL-4 is secreted by activated Th2 lymphocytes, basophils, and mast cells, but not macrophages^17^. In this study, we established macrophages that continuously secrete IL-4, and found that single pulmonary macrophage transplantation (PMT) had an improved therapeutic effect on mice with ALI. Mortality, lung inflammation, and tissue injury decreased in mice transplanted with IL-4-secreting macrophages. PMT has been demonstrated as an effective therapy for lung diseases^18^; nevertheless, its effect on ALI has not been reported. Our study provides practicable strategies for ALI treatment and an application of macrophage transplantation.

## Results

### Establishment of macrophages secreting IL-4

**Fig. 1:**
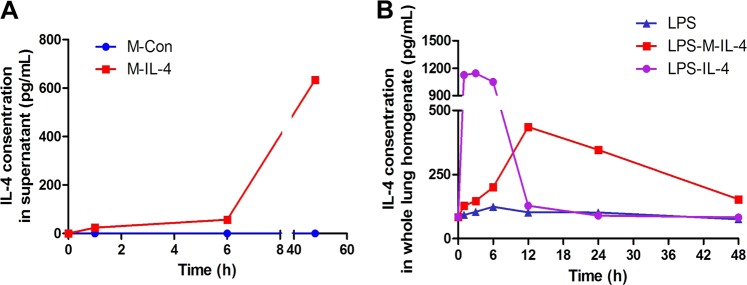
Establishment of murine macrophages secreting IL-4 (M-IL-4). **a** The level of IL-4 in the supernatant of M-IL-4 or M-Con was determined by ELISA. **b** The level of IL-4 in the lung homogenate was determined by ELISA. Mice were treated with LPS (15 mg/kg) and after 0.5 h, PBS, IL-4 (10 ng/g), or M-IL-4 (2.5 × 10^5^ cells/mouse) was intratracheally administered. The lungs were isolated at the indicated time points (*n* = 8 for each group)

Based on the role of macrophages in pulmonary homeostasis and their formidable secretion potential, we constructed macrophages that constitutively express and secrete IL-4 to serve as a stable IL-4 source. The murine monocyte/macrophage cell line, RAW264.7, and bone marrow-derived macrophages (BMDMs) were used to establish a culture of IL-4-secreting macrophages (M-IL-4) and control macrophages (M-Con). IL-4 levels in M-IL-4 supernatant increased in the cell culture and reached 634.25 (±20.45) pg/mL after 48 h. Meanwhile, IL-4 levels in M-Con supernatant were almost undetectable at the three time points (Fig. 1a). IL-4 levels in the lungs of M-IL-4 transplantation mice, IL-4-injected mice, and phosphate-buffered saline (PBS)-injected mice were also detected at different time points (1, 3, 6, 12, 24, and 48 h). The results indicated that IL-4 level strikingly decreased after 6 h of IL-4 injection to mice. In contrast, the level in M-IL-4-transplanted mice increased before 12 h and sustained a relatively high level within 48 h (Fig. 1b). These results indicate that engineered macrophage transplantation should be a viable and sustained IL-4 source in vivo.

### M2-driving ability and the anti-inflammatory effect of M-IL-4

**Fig. 2:**
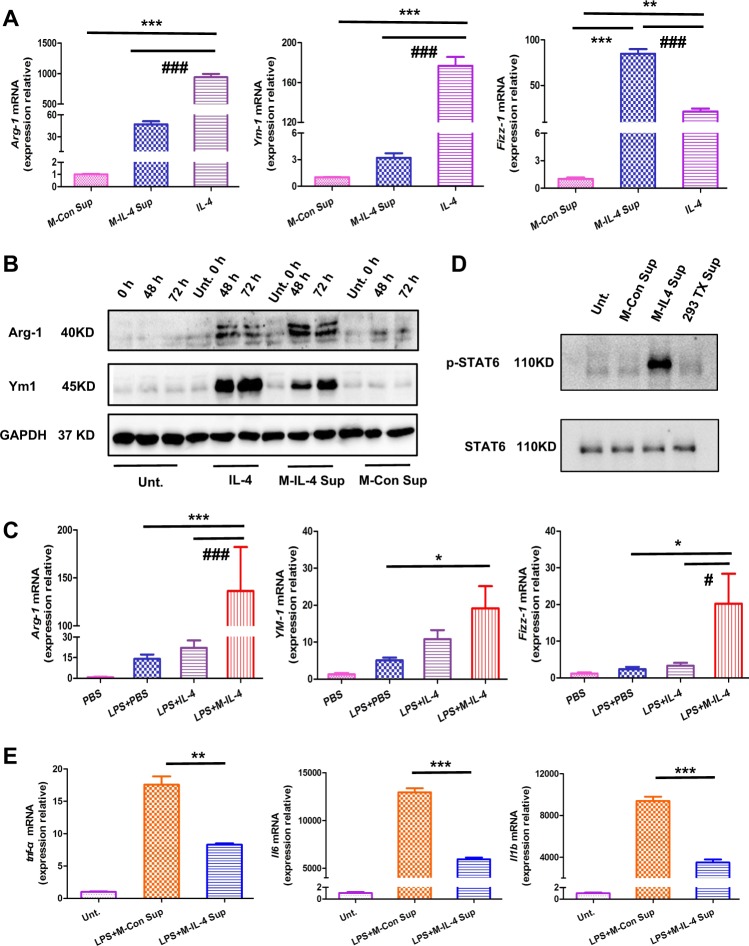
M2-driving ability and the anti-inflammatory effect of murine macrophages secreting IL-4 (M-IL-4). **a** Bone marrow-derived macrophages (BMDMs) were treated with IL-4, M-IL-4 supernatant, or M-Con supernatant for 48 h. Cells were collected and the mRNA level (*Arg-1*, *Ym-1*, and *Fizz-1*) was determined by qPCR. **b** BMDMs were treated with IL-4, M-IL-4 supernatant, or M-Con supernatant and the cells collected at the indicated time. The protein levels of M2 markers (*Arg-1*, and *Ym-1*) were determined by western blotting (WB). **c** Mice were treated with LPS (15 mg/kg) and after 0.5 h, PBS, IL-4 (10 ng/g), or M-IL-4 (2.5 × 10^5^ cells/mouse) was intratracheally transplanted. The lungs were isolated after 48 h (*n* = 6 for each group), and the expression of M2 markers (*Arg-1*, *Ym-1*, and *Fizz-1*) was determined by qPCR. **d** IL-4 and M-IL-4 supernatant activated the STAT6 signaling pathway. BMDMs were treated with M-IL-4 supernatant, M-Con supernatant, 293TX supernatant, or no supernatant, and after 24 h, cells were collected for WB. **e** BMDMs were treated with/without LPS and after 0.5 h, M-IL-4 supernatant or M-Con supernatant was added for 6 h, followed by the collection of cells. The mRNA expression of M1 markers (*tnf-α*, *Il6*, and *Il1b*) was determined. *P < 0.05, **P < 0.01; ^#^*P* < 0.05, ^###^*P* < 0.001, the error bars represent ± SD

We sought to determine whether M-IL-4 could drive M2 polarization of macrophages in vitro and in vivo. Aligning with direct IL-4 treatment, BMDMs treated with M-IL-4 supernatant expressed M2 markers (*Arg-1*, *Ym-1*, and *Fizz-1*) at the mRNA and protein levels to a greater extent (Fig. 2a, b). M-IL-4 also significantly increased the expression of *Arg-1*, *Ym-1*, and *Fizz-1* in mice. *Arg-1* or *Fizz-1* expression in the M-IL-4 treatment group was approximately six- or sevenfold higher than that in the IL-4 or PBS treatment group (Fig. 2c; *Arg-1*, *P* < 0.001; *Fizz-1*, *P* < 0.05); *Ym-1* expression in the M-IL-4 treatment group was approximately twofold higher than in the IL-4 treatment group (Fig. 2c; *Arg-1*, *P* < 0.05). In addition, the STAT6 signaling pathway, which controls M2 polarization in BMDMs, was activated by the M-IL-4 supernatant (Fig. 2d). We also determined the anti-inflammatory effect of M-IL-4. Compared to BMDMs treated with the M-Con supernatant, the expression of *tnf-α*, *ll6*, and *Il1b* in BMDMs treated with M-IL-4 supernatant was significantly reduced after LPS treatment (Fig. 2e, *P* < 0.001). These results suggest that M-IL-4 can inhibit M1 activation in other macrophages and promote M2 polarization in vitro and in vivo by secreting IL-4.

### M-IL-4 transplantation alleviates lung injury

**Fig. 3:**
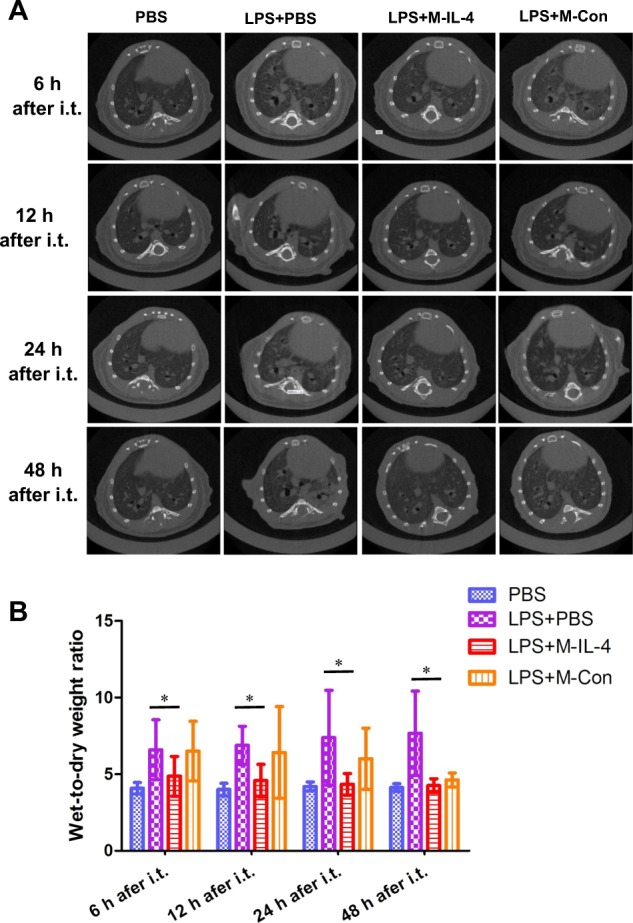
Microcomputed tomography and wet/dry ratio show transplantation of murine macrophages secreting IL-4 (M-IL-4) alleviates lung injury in mice. Mice were exposed to LPS (15 mg/kg). After 0.5 h, PBS, M-IL-4 (2.5 × 10^5^ cells/mouse), or M-Con (2.5 × 10^5^ cells/mouse) was intratracheally transplanted (*n* = 8 for each group). **a** Microcomputed tomography (micro-CT) images are presented; and the wet/dry ratio (**b**) were determined; *P < 0.05; **P < 0.01, the error bars represent ± SD

**Fig. 4:**
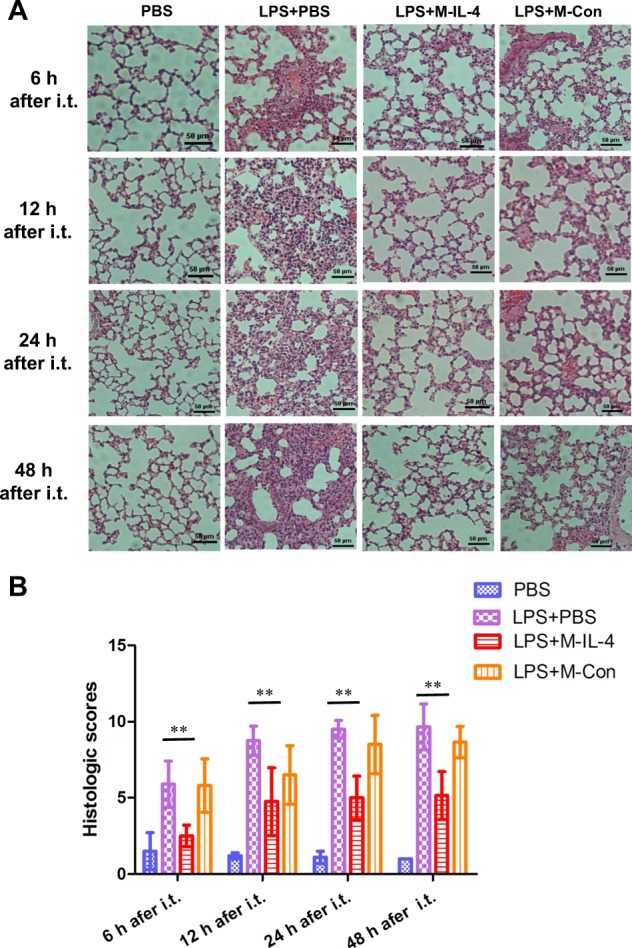
Histopathological observation and lung injury scores indicate that transplantation of murine macrophages secreting IL-4 (M-IL-4) decreases lung injury in mice. Mice were exposed to LPS (15 mg/kg). After 0.5 h, PBS, M-IL-4 (2.5 × 10^5^ cells/mouse), or M-Con (2.5 × 10^5^ cells/mouse) was intratracheally transplanted (*n* = 8 for each group). **a** The lungs were collected and stained with HE for histological examination, and the lung injury index (**b**) was assessed; *P < 0.05; **P < 0.01, the error bars represent ± SD

To clearly reveal the extent of lung damage and the effect of M-IL-4 treatment, micro-CT scanning and HE staining were respectively employed. M-IL-4 or M-Con cells were transplanted 0.5 h after LPS administration. One three-dimensional image per mouse was captured to analyze the internal inflammatory response of whole lung after 6, 12, 24, and 48 h (*n* = 8 per group at each time point). Overall, the lungs of mice challenged with LPS developed more inflammation over time (Fig. 3a), and evidently, M-IL-4 decreased the area of lung inflammation based on the image obtained. While whole left lung was used for histopathology, the right lung was used to evaluate wet-to-dry (W/D) weight ratio at 6, 12, 24, and 48 h after LPS injection. Lung inflammation was aggravated at 48 h after LPS injection in all treated mice compared to the PBS group (Fig. 4a); M-IL-4 transplantation reduced the extent of lung inflammation, aligning with the results of micro-CT scanning. Moreover, M-IL-4 transplantation led to a statistically significant decrease in lung W/D ratio at 6, 12, 24, and 48 h (Fig. 3b) in treated mice than in PBS control mice after LPS injection. However, the M-Con transplanted group also had a lower W/D ratio, and there was no significant difference between the M-Con transplanted group and the LPS injection group (*P* > 0.05). To quantify lung injury, we determined the effect of M-IL-4 transplantation on LPS-induced lung injury index. Indeed, the histological scores of lung injury index in M-IL-4-transplanted mice were significantly decreased compared to those in the PBS-treated mice at 6, 12, 24, or 48 h after LPS injection (*P* < 0.01) (Fig. 4b). There was no significant difference in the lung injury index between M-Con transplanted group and the PBS group (*P* > 0.05).

### M-IL-4 transplantation attenuates LPS-induced lung inflammation

**Fig. 5:**
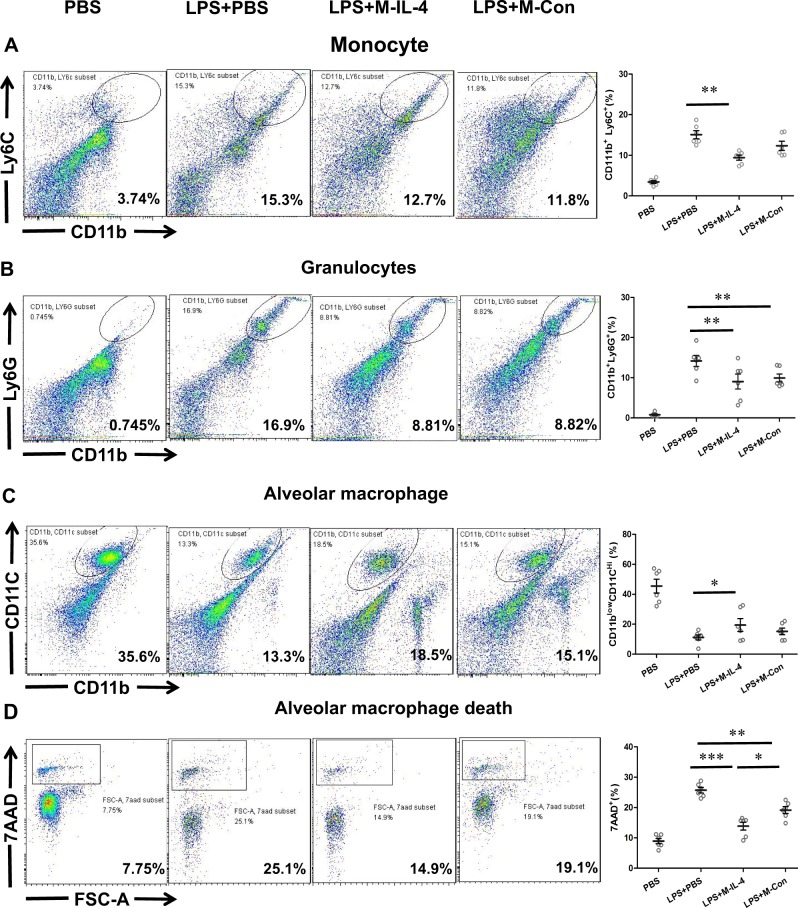
Transplantation of murine macrophages secreting IL-4 (M-IL-4) decreases infiltration of inflammatory cells and alveolar macrophage death. Mice were exposed to LPS (15 mg/kg). After 0.5 h, PBS, M-IL-4 (2.5 × 10^5^ cells/mouse), or M-Con (2.5 × 10^5^ cells/mouse) was intratracheally transplanted (*n* = 6 for each group). The BALF was collected at 6 h after LPS administration, and the BAL cells were used to analyze by flow cytometry. **a** CD11b^+^ and Ly6C^+^ (monocyte) BAL cells identified by flow cytometry; **b** CD11b^+^ and Ly6G^+^ (granulocytes) BAL cells identified by flow cytometry; **c** CD11b^low^ and CD11C^hi^ (alveolar macrophage) BAL cells identified by flow cytometry; **d** Alveolar macrophage stained with 7AAD were measured by flow cytometry. *P < 0.05; **P < 0.01, the error bars represent ± SD

**Fig. 6:**
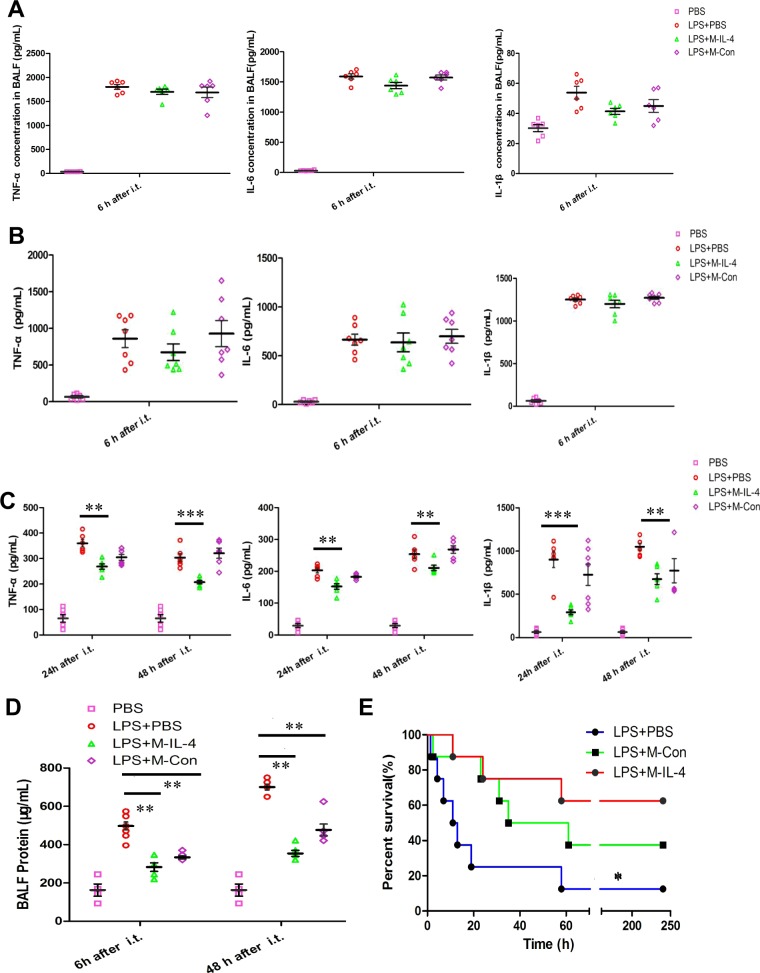
Transplantation of murine macrophages secreting IL-4 (M-IL-4) attenuates lung inflammation in mice. Mice were exposed to LPS (15 mg/kg). After 0.5 h, PBS, M-IL-4 (2.5 × 10^5^ cells/mouse), or M-Con (2.5 × 10^5^ cells/mouse) was intratracheally transplanted (*n* = 8 for each group). The lungs were isolated at the indicated time points (*n* = 8 for each group). **a** The levels of TNF-α, IL-6, IL-1β in BALF at 6 h after LPS injection was determined by ELISA. **b**, **c** The levels of pro-inflammatory factors (TNF-α; IL-6; IL-1β) in lung homogenate at 6 h, 24 h and 48 h after LPS injection were determined by ELISA. **d** Total protein in BALF was determined by BCA protein assay (*n* = 6 for each group). **e** Mice were exposed to LPS (20 mg/kg). After 0.5 h, PBS, M-IL-4 (2.5 × 10^5^ cells/mouse), or M-Con (2.5 × 10^5^ cells/mouse) was intratracheally transplanted (*n* = 8 for each group), and Kaplan–Meier survival curves for mice are presented. *P < 0.05; **P < 0.01, the error bars represent ±SD

A previous study showed that alveolar macrophages play a major role in LPS-induced inflammatory cells accumulation in the lungs^19^. We wondered whether monocyte, granulocyte, and alveolar macrophages in BAL cells and pro-inflammatory cytokines in the BALF were difference at 6 h after M-IL-4 or M-Con transplantation in ALI mice. BAL cells were isolated and analyzed by flow cytometry for CD11b^+^Ly6C^+^ (monocytes), CD11b^+^Ly6G^+^ (granulocytes), and CD11b^low^CD11C^hi^ (alveolar macrophages). The results showed that the percentage of monocytes and granulocytes increased in the BAL cells after LPS instillation (Fig. 5a, b), whereas the M-IL-4 cells significantly decreased the percentage of monocytes and granulocytes (*P* < 0.01). M-Con cells also significantly decreased the percent of granulocytes (*P* < 0.01, Fig. 5b). The percentage of alveolar macrophages decreased 6 h after LPS instillation compared to the PBS control group (Fig. 5c), and the reduction ratio of alveolar macrophages in the M-IL-4-transplanted group was lower than the LPS + PBS group (*P* < 0.05, Fig. 5c). Next, we examined whether LPS could induce alveolar macrophages necrosis and whether M-IL-4 could reduce AM necrosis. 7AAD was used to detect necrotic cells in the CD11b^low^CD11C^hi^ (alveolar macrophages) cells population. Following LPS administration, alveolar macrophages underwent increased necrotic cell death, and M-IL-4 cells and M-Con cells both significantly reduced necrotic cell death (*P* < 0.01, Fig. 5d). Reduced necrotic cell death from M-IL-4 cells was more significant than M-Con cells (*P* < 0.05, Fig. 5d). In addition, LPS increased the secretion of pro-inflammatory cytokines (TNF-α, IL-6, IL-1β) in the BALF; however, M-IL-4 transplantation did not significantly reduce the levels of TNF-α, IL-6, and IL-1β in the BALF (Fig. 6a). These data show that M-IL-4 can attenuate LPS-induced lung inflammation, and that this may be related to reduced infiltration of inflammatory cells and alveolar macrophage death.

To elucidate the anti-inflammatory effect of M-IL-4 in vivo, we transplanted M-IL-4 cells into the lungs of mice and assessed the lung inflammatory factors. M-IL-4 transplantation did not significantly alter the secretion of TNF-α, IL-6, and IL-1β secretion at 6 h after administration (Fig. 6b), but notably reduced their secretions after 24 and 48 h (Fig. 6c). Meanwhile, M-IL-4 transplantation resulted in a significant reduction in the BALF protein level (Fig. 6d). These results suggest that M-IL-4 possesses anti-inflammatory activity and can sustain pulmonary macrophages with the M2 phenotype in vivo.

### M-IL-4 transplantation significantly improves the survival of mice with ALI

Finally, we investigated whether M-IL-4 transplantation could improve the survival rate of mice with ALI. Compared to the control mice after LPS exposure, M-IL-4-transplanted mice demonstrated a significant improvement in their survival rates (Fig. 6e; 62.5% vs. 12.5%, 62.5% vs. 7.5%, *P* < 0.05). These results demonstrate that M-IL-4 transplantation was therapeutically effective in ALI.

## Discussion

As macrophages are crucial for the initiation and resolution of ALI, they are a potential therapeutic target in ALI. In this study, we established engineered macrophages expressing and secreting IL-4, an anti-inflammatory cytokine, and found that single PMT had an improved therapeutic effect on mice with ALI. To our knowledge, this is the first study to report the application of macrophage transplantation in the treatment of ALI.

Although direct and repeated IL-4 treatment has been demonstrated as an effective therapeutic method for ALI^20–22^, PMT has significant advantages. On the one hand, only a single intratracheal injection is needed in PMT. However, in direct IL-4 cytokine treatment, repeated injections must be performed, which could result in tracheal injury and possibly, a new lung infection. Due to the function of the M2 phenotype of transplanted macrophages, they can directly eliminate inflammation and promote tissue repair^23^; thus, the acute phase of ALI could be terminated early, and the resolving phase initiated immediately after transplantation. This rapid switch in phase would be beneficial for the ALI recovery.

When the pathogen is cleared, lung inflammation is not immediately terminated, and pro-inflammatory signaling caused by foreign antigens or host-derived alarms may not be sustained. In fact, resolution of lung inflammation and return to tissue homeostasis is an active, tightly coordinated process which reverses all of the steps involved in initiation of the inflammatory response and induces counter-regulatory mechanisms^23^. Engineered RAW264.7 cells can secrete IL-4, which can induce M2 polarization of macrophages^14^. Some research has shown that more M2 macrophages can induce pulmonary fibrosis^24^, airway hyper reactivity^25^, and tumor cell migration^26^. RAW264.7 cells are derived from BALB/C, thus we transplanted those into C57BL/6J mice. We hoped that engineered RAW264.7 cells were excluded by heterogeneous rejection ultimately.

The limitations of our study include the absence of a minimum effective cell dose, maximum tolerated dose, or the best time point to transplant after LPS injection. Moreover, the location and dynamic change of transplanted cells remain as unresolved issues; thus, additional studies are needed to confirm the precise pharmacokinetics and durability of the engineered macrophages. Additionally, persistent IL-4 exposure would be harmful; therefore, an inducible expression system and/or a turnoff strategy should be developed and implemented in future investigations. Finally, an M-IL-4 plus IL-4 therapeutic strategy should be employed to account for the lack of immediacy in cell transplantation and ameliorate the unsustainability of cytokine treatment.

## Materials and methods

### Animals and models

Female, healthy, C57BL/6J mice (7–8 weeks old, specific pathogen free) were purchased from Beijing Huafukang Bioscience Co. Inc. (Beijing, China). The experiments were conducted using a protocol approved by the Chinese Academy of Military Medicine Science Animal Care and Use Committee (Beijing, China).

As performed in previous studies^15,27–29^, mice were anesthetized with intraperitoneal ketamine (220 mg/kg) prior to exposure of the trachea. The ALI mouse model was induced by lipopolysaccharide (LPS, L2880; *Escherichia coli* 055:B5; Sigma-Aldrich, St. Louis, USA). Briefly, the mice were given LPS (15, 20 mg/kg mouse weight diluted in PBS, 50 μL) intratracheally, while control mice were intratracheally administered 50 μL of sterile PBS.

### Cell culture

Primary BMDMs were obtained from 6- to 8-week-old C57BL/6J mice by isolating and flushing the tibias and femurs. BMDMs were cultured with DMEM (Gibco) supplemented with 10% FBS, 30% L929-conditioned media (LCM), and 1% penicillin/streptomycin (pen/strep). Murine macrophage RAW264.7 cells, conserved in our laboratory, were cultured in RPMI 1640 medium containing 10% FBS and 1% pen/strep. BMDMs and RAW264.7 cells were maintained at 37 °C in a 5% CO_2_ humidified atmosphere. RAW264.7 cells were previously transplanted in mice to cure neurodegenerative diseases^30^.

### Vector constructs and lentivirus transfection

The lentiviral constructs expressing mouse IL-4 were cloned into the pCDH vector (SBI). IL-4 cDNA was obtained from the National Center for Biotechnology Information. Lentiviralvectors were transfected with packing plasmids into 293T cells for 2 days, and the viral particles were used to infect RAW264.7 cells and BMDMs. Selection was carried out by culturing cells in medium containing 2 μg/mL puromycin for 2 days. RAW264.7 cells with IL-4 were marked as M-IL-4, and non-IL-4 cells were marked as M-Con.

### Whole lung homogenate RNA isolation

Whole-lung tissues were homogenized in PBS (g/v = 1:10), and 200 µL of the lung homogenate was used to isolate total RNA using the RNAprep pure Tissue Kit (Tiangen, China) according to the manufacturer’s protocol. Total RNA was used to synthesize cDNA using PrimeScript TM RT Master Mix (Takara, Japan).

### Real-time PCR analysis

Target gene expression (*tnf-α*, *Il6*, and *Il1b*) was detected by reverse transcriptase PCR (RT-PCR) on an Agilent Mx3005P qPCR System (Agilent, USA). Target gene expression levels were normalized to the housekeeping gene, *β-actin*, and fold change was calculated using the 2^−ΔΔCT^ method.

The primers used for real-time RT-PCR were: *tnf-α*, forward 5′-GACCCTCACACTCAGATCATC-3′ and reverse 5′-GAACCTGG GAGTAGATAAGG-3′; *Il6*, forward 5′-TGAACAACGATGATGCAC TTGC-3′ and reverse 5′-GTACTCCAGAAGACCAGAGGAAAT-3′; *Il1b*, forward 5′-CTCCATGAGCTTTGTACAAGG-3′ and reverse 5′-TGCTGATGTACCAGTTGGGG-3′; and *β-actin*, forward 5′-TTGTTAC CAACTGGGACG-3′ and reverse 5′-CCAGAGGCATACAGG GAC-3′.

### ELISA

The levels of cytokines from the supernatant of whole-lung homogenate were measured using commercially available mouse TNF-α, IL-6, and IL-1β ELISA kits (R&D Systems Inc., Minneapolis, MN, USA). The experiment was repeated three times, and the results are presented as the mean value.

### Bronchoalveolar lavage fluid analysis

Mice were subjected to bronchoalveolar lavage (BAL) to collect BAL fluid (BALF) as described previously^31^. The trachea was exposed and cannulated with a catheter. Whole lung was lavaged three times with sterile PBS in a volume of 0.8 mL/wash; on average, the fluid recovered after lavage was greater than 80%. The BALF was centrifuged at 3000 r.p.m. for 20 min at 4 °C, and the supernatant was collected and frozen at −80 °C to assay total protein using the BCA Protein Assay Kit (Tiangen, China) and the level of cytokines (TNF-α, IL-6, and IL-1β) according to the manufacturer’s protocol. The BAL cells were used to analyze cell type.

### Flow cytometry

BAL cells were isolated through a 70-µm cell strainer (Biologix Group Limited). After washing with PBS and resuspending with 0.5% bovine serum albumin in PBS, cells were stained with flow cytometry antibodies to detect CD11b (eBioscience), CD11C (eBioscience), Ly6C (eBioscience), and Ly6G (eBioscience) according to the manufacturer’s instructions. In addition, 7AAD was used to detect necrotic cells. Data were acquired using a FACS Aria II flow cytometers (BD Biosciences), and the results were analyzed using FlowJo software (Version 7.6).

### Western blot analysis

Proteins were extracted using RIPA buffer and total protein was quantified using a BCA protein assay reagent kit (Tiangen, China). Equal amounts of proteins were separated on a 12% SDS-PAGE gel and transferred onto a PVDF membrane (PALL, USA) prior to incubation with the primary antibodies, anti-Arg-1 (Cell Signaling Technology, USA), anti-YM-1 (Cell Signaling Technology, USA), and anti-GAPDH (Santa Cruz Biotechnology, USA) 12 h at 4 °C (antibodies diluted 1:1000 in blocking solution). After three washes in 1× TBST buffer, the blots were incubated with either horseradish peroxidase-conjugated goat anti-rabbit antibody or horseradish peroxidase-conjugated goat anti-mouse antibody (1:2000; Jackson Immuno Research, USA) for 1 h at 25 °C, re-washed, and then developed using an ECL chemiluminescence kit (Thermo Fisher Scientific, USA).

### Histopathological observation and lung injury scores

Morphological analysis of mouse lung inflammation was performed by micro-CT scanning (Quantum FX Demo, PerkinElmer-Caliper LS, MA, USA) and hematoxylin–eosin (HE) staining. Whole left lower lobe of the lung was fixed in 4% formaldehyde neutral buffer solution for 48 h, dehydrated in a graded ethanol series, embedded in paraffin, and sliced into 5-µm sections. These sections were then stained with HE for histopathological analysis.

Two pathologists blinded to the treatment regimen were invited to assess the severity of lung injury using the following histological features: edema, hyperemia and congestion, neutrophil margination and tissue infiltration, intra-alveolar hemorrhage and debris, and cellular hyperplasia. A scoring system was used to grade the degree of lung injury.

Each feature was graded as absent, mild, moderate, or severe, and was assigned a score from 0 to 3. Total score was calculated for each mouse^27^.

### Lung W/D weight ratio

The superior lobe of the right lung was cleansed and weighed to obtain the wet weight. The section was then placed in an oven at 80 °C for 24 h to measure the dry weight. The ratio of W/D weight was calculated to assess tissue edema^1^.

### Statistical analyses

All values are reported as mean ± SE. Parametric or nonparametric testing was performed as indicated. Kaplan–Meier survival curves were assessed by a log-rank Mantel–Cox test. Student’s *t*-test was used for all other analyses when two groups were compared at one time point, or one-way ANOVA with Tukey’s post-hoc test when multiple groups were compared at one time point. *P* < 0.05 was used as the cut-off point for significance; *P < 0.05, **P < 0.01, and ***P < 0.001.

## References

[1] Zhao YF (2015). Mesenchymal stem cell-based FGF2 gene therapy for acute lung injury induced by lipopolysaccharide in mice. Eur. Rev. Med. Pharm. Sci..

[2] Fan EKY, Jie F (2018). Regulation of alveolar macrophage death in acute lung inflammation. Respir. Res..

[3] Liu Q, Li W, Zeng QS, Zhong NS, Chen RC (2013). Lung stress and strain during mechanical ventilation in animals with and without pulmonary acute respiratory distress syndrome. J. Surg. Res..

[4] Malcolm L, Jihad M, Didier T (2013). Prone positioning in the acute respiratory distress syndrome. New Engl. J. Med..

[5] Guangxi L (2011). Eight-year trend of acute respiratory distress syndrome: a population-based study in Olmsted County, Minnesota. Am. J. Respir. Crit. Care Med..

[6] Dreymueller D (2012). Lung endothelial ADAM17 regulates the acute inflammatory response to lipopolysaccharide. EmBO Mol. Med..

[7] Matutebello G, Frevert CW, Martin TR (2008). Animal models of acute lung injury. Am. J. Physiol. Lung Cell. Mol. Physiol..

[8] Johnston LK, Rims CR, Gill SE, Mcguire JK, Manicone AM (2012). Pulmonary macrophage subpopulations in the induction and resolution of acute lung injury. Am. J. Respir. Cell. Mol. Biol..

[9] Geasorlí S, Guillamat R, Serranomollar A, Closa D (2011). Activation of lung macrophage subpopulations in experimental acute pancreatitis. J. Pathol..

[10] Dhaliwal K (2012). Monocytes control second-phase neutrophil emigration in established lipopolysaccharide-induced murine lung injury. Am. J. Respir. Crit. Care Med..

[11] Odegaard JI, Chawla A (2011). Alternative macrophage activation and metabolism. Annu. Rev. Pathol..

[12] Arranz A (2012). Akt1 and Akt2 protein kinases differentially contribute to macrophage polarization. Proc. Natl Acad. Sci. USA.

[13] Sindrilaru A (2011). An unrestrained proinflammatory M1 macrophage population induced by iron impairs wound healing in humans and mice. J. Clin. Invest..

[14] Kellywelch AE, Hanson EM, Boothby MR, Keegan AD (2003). Interleukin-4 and interleukin-13 signaling connections maps. Science.

[15] D’Alessio FR (2016). Enhanced resolution of experimental ARDS through IL-4-mediated lung macrophage reprogramming. Am. J. Physiol. Lung Cell. Mol. Physiol..

[16] Gao S (2015). Curcumin induces M2 macrophage polarization by secretion IL-4 and/or IL-13. J. Mol. Cell. Cardiol..

[17] Hou J (1994). An interleukin-4-induced transcription factor: IL-4 Stat. Science.

[18] Suzuki T (2014). Pulmonary macrophage transplantation therapy. Nature.

[19] Harmsen AG (1988). Role of alveolar macrophages in lipopolysaccharide-induced neutrophil accumulation. Infect. Immun..

[20] Bao L, Alexander JB, Shi VY, Mohan GC, Chan LS (2015). Interleukin-4 up-regulation of epidermal interleukin-19 expression in keratinocytes involves the binding of signal transducer and activator of transcription 6 (Stat6) to the imperfect Stat6 sites. Immunology.

[21] Finkelman FD (1993). Anti-cytokine antibodies as carrier proteins. Prolongation of in vivo effects of exogenous cytokines by injection of cytokine-anti-cytokine antibody complexes. J. Immunol..

[22] Bosurgi L (2017). Macrophage function in tissue repair and remodeling requires IL-4 or IL-13 with apoptotic cells. Science.

[23] Herold S, Mayer K, Lohmeyer J (2011). Acute lung injury: how macrophages orchestrate resolution of inflammation and tissue repair. Front. Immunol..

[24] Gharib SA (2014). MMP28 promotes macrophage polarization toward M2 cells and augments pulmonary fibrosis. J. Leukoc. Biol..

[25] Kaur M, Bell T, Salek-Ardakani S, Hussell T (2015). Macrophage adaptation in airway inflammatory resolution. Eur. Respir. Rev..

[26] Jeffrey W (2004). A paracrine loop between tumor cells and macrophages is required for tumor cell migration in mammary tumors. Cancer Res..

[27] Ohsawa I (2007). Hydrogen acts as a therapeutic antioxidant by selectively reducing cytotoxic oxygen radicals. Nat. Med..

[28] Wang BQ (2019). Knockdown of TFPI-anchored endothelial cells exacerbates lipopolysaccharide-induced acute lung injury via NF-κB signaling pathway. Shock.

[29] Liu H (2015). Combination therapy with nitric oxide and molecular hydrogen in a murine model of acute lung injury. Shock.

[30] Haney MJ (2013). Specific transfection of inflamed brain by macrophages: a new therapeutic strategy for neurodegenerative diseases. Plos One.

[31] Bhandari V (2006). Hyperoxia causes angiopoietin 2-mediated acute lung injury and necrotic cell death. Nat. Med..

